# Neural Substrates of Cognitive Subtypes in Parkinson's Disease: A 3-Year Longitudinal Study

**DOI:** 10.1371/journal.pone.0110547

**Published:** 2014-10-20

**Authors:** Yumiko Shoji, Yoshiyuki Nishio, Toru Baba, Makoto Uchiyama, Kayoko Yokoi, Toshiyuki Ishioka, Yoshiyuki Hosokai, Kazumi Hirayama, Hiroshi Fukuda, Masashi Aoki, Takafumi Hasegawa, Atsushi Takeda, Etsuro Mori

**Affiliations:** 1 Department of Behavioral Neurology and Cognitive Neuroscience, Tohoku University School of Medicine, Sendai, Japan; 2 Department of Speech, Language and Hearing Sciences, Niigata University of Health and Welfare, Niigata, Japan; 3 Department of Occupational Therapy, Saitama Prefectural University, Koshigaya, Japan; 4 Department of Diagnostic Image Analysis, Tohoku University School of Medicine, Sendai, Japan; 5 Department of Occupational Therapy, Yamagata Prefectural University of Health Science, Yamagata, Japan; 6 Department of Radiology and Nuclear Medicine, Institute of Development, Aging, and Cancer, Tohoku University, Sendai, Japan; 7 Department of Neurology, Tohoku University School of Medicine, Sendai, Japan; 8 Department of Neurology, Sendai Nishitaga Hospital, Sendai, Japan; Nathan Kline Institute and New York University School of Medicine, United States of America

## Abstract

**Background:**

The neuropsychological features and neuropathological progression patterns associated with rapidly evolving cognitive decline or dementia in Parkinson's disease (PD) remain to be elucidated.

**Methods:**

Fifty-three PD patients without dementia were recruited to participate in a 3-year longitudinal cohort study. The patients were grouped according to the Clinical Dementia Rating (CDR). Group-wise comparisons were made with regard to demographic characteristics, motor symptoms, neuropsychological performances and 18F-fluorodeoxyglucose positron emission tomography.

**Results:**

Patients who had memory-plus cognitive impairment (patients whose CDR was 0 at baseline and 0.5 in memory and other domains at follow-up, and those whose baseline CDR was 0.5 in memory and other domains) exhibited higher age at onset, visuoperceptual impairment, non-tremor-dominant motor disturbance, rapid symptomatic progression and posterior neocortical hypometabolism. In patients who were cognitively unimpaired and those who had memory-dominant cognitive impairment (patients whose CDR was 0 at baseline and 0.5 only in memory domain at follow-up, and those whose baseline CDR was 0.5 only in memory domain), the posterior neocortex was relatively unaffected until a later stage of the disease.

**Conclusions:**

These results suggest that visuoperceptual impairment and the early involvement of the posterior neocortex may be risk factors for rapid symptomatic progression and dementia in PD.

## Introduction

The cognitive features of Parkinson's disease (PD) are heterogeneous and can be categorized into several major subtypes. [1], [2] However, the neural substrates underlying the cognitive subtypes remain to be elucidated. Recent studies have demonstrated that there are correlations between cognitive impairment and non-cognitive features in PD: patients who develop dementia have a higher age of onset, rapid symptomatic progression, anosmia and a non-tremor-dominant motor subtype. [3], [4], [5], [6] Consistent with these observations, neuropathological studies have suggested that the anatomical distribution of Lewy-related pathology differs depending on the clinical subtypes. The pathology rapidly evolves from the brainstem into the cerebral cortex in patients with the non-tremor-dominant motor subtype and/or dementia, whereas it is relatively confined to the brainstem for a longer period of time in patients with a tremor-dominant motor subtype and no cognitive impairment. [7] If such provisional clinico-pathological relationships are genuine and if specific subtypes of cognitive impairment are associated with the future development of dementia, these cognitive subtypes may be associated with specific clinico-pathological subtypes.

Previous morphometric MRI and 18F-fluorodeoxyglucose positron emission tomography (FDG-PET) studies have demonstrated greater frontal, temporal and occipital gray matter volume reduction and greater frontal and parietal cortical hypometabolism in PD patients with dementia or mild cognitive impairment (MCI) compared with cognitively unimpaired patients. [8], [9], [10], [11], [12] In agreement with these neuroimaging findings, several neuropathological studies demonstrated the relationship between dementia and limbic and/or neocortical neurodgeneration. [13], [14], [15] However, there is only a little evidence for neuroimaging features predictive of later development of dementia and for distinctive progression patterns of cortical lesions among the PD subtypes. The sole previous longitudinal FDG-PET study of PD demonstrated that patients who developed dementia 1 to 3 years later exhibited occipito-parietal hypometabolism at baseline. [16] To further address this issue, we investigated the relationship among cognitive subtypes, other clinical features and changes in regional brain glucose metabolism (CMRglc) over 3 years in a cohort of PD patients.

## Methods

All procedures in this study followed the clinical study guidelines of Tohoku University Hospital and were approved by its ethics committee. The patients gave written informed consent after receiving a detailed explanation of the study. When the patients had a compromised ability to consent, their family members gave consent on behalf of the patients.

### Subjects

We analyzed 55 patients with PD without dementia (mean age 65.4±6.5 years; 27 women) who participated in a 3-year longitudinal study at Tohoku University Hospital. Details of the study design have been described elsewhere. [3], [9], [17] Briefly, outpatients at the movement disorder clinic who met the following criteria were recruited in the study: fulfillment of the diagnostic criteria of the United Kingdom Parkinson's Disease Society Brain Bank; aged 50 years or more; absence of dementia according to the Diagnostic Statistical Manual-IIIR [18] and a Clinical Dementia Rating (CDR) [19] overall score of 0 or 0.5, no evidence of diabetes mellitus; no history of other neurological or psychiatric diseases; and no evidence of infarcts, bleedings, tumors and other focal brain lesions on MRI. Of 88 consecutive patients, 33 patients dropped out for the following reasons: 4 patients died; 4 were institutionalized; 1 developed psychosis; 2 developed myocardial infarction or cerebral infarction; 9 moved to hospitals near their homes; 6 did not return for follow-up visits for unknown reasons; the initial diagnosis of PD was dismissed in 3 patients; and 4 were excluded because of incomplete clinical or imaging data. Fourteen healthy volunteers (mean age 63.1±4.4 years; 6 women) were recruited as controls for neuroimaging. There were no significant differences in age (*t* = 1.6, p = 0.1) or sex (x^2^ = 0.2, p = 0.7) between the patient and control groups.

### Comparison of patient classification procedures: the neuropsychology-based criteria versus the Clinical Dementia Rating

Measuring cognitive changes is challenging because there is no very reliable change measures. Practice effects associated with the repeated administration have a great impact on neuropsychological test performance, yielding spurious cognitive improvement over time. [20], [21], [22], [23] A recent study demonstrated that previous test exposures lead to bias towards normal cognition in the diagnosis of MCI. [24]In addition, cognitive assessment in PD is complicated by motor symptoms, such as bradykinesia and tremor, and medication-related effects. [2] To take these problems into account, global cognitive measures and/or caregiver interviews have been used in longitudinal intervention trials for cognitive disorders. [25], [26], [27], [28] According to this convention, we have introduced the CDR, a global cognitive measure based on examinations by clinicians and caregiver interview, in our cohort study of PD. [3], [9], [17] To examine the rationality of the use of the CDR in the classification of cognitive status in PD, we compared the 3-year cognitive changes based on the neuropsychology-based criteria for MCI in PD (PD-MCI) and those based on the CDR in the patients (n = 46) who completed neuropsychological tests for memory, visuoperceptual ability and attention/working memory (see below for the details of the neuropsychological tests). PD-MCI was defined according to the Movement Disorder Society Guideline for PD-MCI Level I (MDS PD-MCI criteria), in which the diagnosis of PD-MCI required impairments of 1 to 2 standard deviations (SDs) below norms on at least 2 neuropsychological tests. [29] In the CDR-based criteria, the patients were classified as CDR 0 (unimpaired cognition) or CDR 0.5 (cognitive impairment which mildly affecting their everyday life).

### Patient classification based on the Clinical Dementia Rating

The CDR, which was designed to provide a rating scale for subjects from normal cognition through various stages of dementia, is widely considered to be a reliable scale for staging the severity of cognitive dysfunction. [19] The CDR comprises 6 subdomains, i.e., memory, orientation, judgment and problem solving, community affairs, home and hobbies and personal care. In matters related to the domains of community affairs, home and hobbies, and personal care, we asked the patients and their caregivers about cognition-related functional decline separately from disability arising from physical impairment in order to eliminate as far as possible the effects of non-cognitive symptoms. [9], [17].

The primary aim of the current study is to discover clinical features and distinctive brain metabolic patterns of patients who have rapid cognitive deterioration. To this end, we first focused on 40 patients who were cognitively unimpaired (CDR 0) at baseline. Among these patients, 26 patients were cognitively unchanged over 3 years (CDR 0 at the third year; non-converters), 7 worsened only in the memory domain (memory-only converters) and 6 worsened in the memory and non-memory domains (memory-plus converters). The remaining patient, who showed deterioration only in a non-memory domain, was excluded from the analyses. Second, we analyzed patients whose overall CDR scores were 0.5 at baseline to investigate longitudinal brain metabolic changes after PD patients developed mild cognitive deficits. Eight patients who scored ≥0.5 only in the memory domain at baseline (baseline memory-only) and 6 patients who scored ≥0.5 in the memory and other domains (baseline memory-plus) were recruited for the study. We speculated that the baseline memory-only and the baseline memory-plus patients may represent the clinico-pathological stages following the memory-only converters and the memory-plus converters, respectively. We conducted group comparisons separately among the groups of baseline CDR 0, specifically non-converter, memory-only converter and memory-plus converter patients, and between the groups of baseline CDR 0.5, specifically baseline memory-only and baseline memory-plus patients, because our interest was in longitudinal changes in clinical symptoms and brain glucose metabolism.

### Cognitive and motor assessments

The Mini-Mental State Examination (MMSE) and the Word Recall subtest of the Alzheimer’s Disease Assessment Scale (ADAS) were used to assess general cognitive function and episodic memory, respectively. [30], [31] Visuoperception was assessed using the correct response score on the overlapping-figure identification test. [32] A subset of patients underwent the backwards digit-span test to assess their working memory (the number of patients is indicated in **Tables 1** **and** **2**). [29] Further details have been described elsewhere. [17], [32] Motor symptoms were assessed using the Unified Parkinson’s Disease Rating Scale (UPDRS) part III. We calculated the rate of progression indices for the clinical measures described above using the following formula: (rate of progression) = [(third year score)-(baseline score)]/(years of interval). [33] The tremor and non-tremor motor scores were calculated based on the UPDRS parts II and III. [5].

**Table 1:** Demographic and clinical profiles of patients with a Clinical Dementia Rating of 0 at baseline.

		Non-converter(N = 26)		Memory-only converter(N = 7)		Memory-plus converter(N = 6)		Differences among groups		
**Age at baseline** (years)		62.2±5.9		67.7±5.5		71.8±2.6		Memory-plus>Non-converters^b^		
**Gender** (male/female)		12/14		2/5		1/5				
**Education** (years)		11.8±2.5		11.1±2.5		12.0±2.8				
**Test-retest interval** (days)		1140.2±110.7		1107.7±43.5		1109.7±59.7				
**Disease duration at baseline** (years)		4.3±3.7		5.0±6.9		5.0±3.2				
**Age at onset** (years)		58.0±7.3		63.6±6.0		67.2±5.4		Memory-plus>Non-converters^b^		
**Levodopa equivalent dose at baseline** (mg/day)		303.5±233.1		378.9±320.4		533.6±340.2				
**UPDRS part III**	Baseline	18.0±7.3		18.9±8.0		16.5±6.2				
	Progression rate(/years)	−0.02±2.0		−0.8±0.8		4.0±5.2		Memory-plus>Non-converters^b^; Memory-plus>Memory-only^b^		
**UPDRS tremor score** ¶	Baseline	0.5±0.4		0.4±0.6		0.3±0.4				
	Third year	0.3±0.3		0.2±0.2		0.3±0.3		Main effect of non-tremor score: Memory-plus>Non-converters^b^;Memory-plus>Memory-only^b^		
**UPDRS non-tremor score** ¶	Baseline	0.7±0.3		0.7±0.4		0.7±0.2				
	Third year	0.8±0.3		0.7±0.4		1.6±0.2				
**CDR sum of boxes**	Baseline	0		0		0		NE		
	Third year	0		0.5		1.8±0.8		NE		
**MMSE**	Baseline (/30)	28.2±1.8		27.3±2.6		27.5±1.9				
	Progression rate(/years)	0.1±0.6		−0.1±0.9		−0.6±0.7				
**ADAS word recall** †	Baseline (/30)	19.3±3.4		17.3±4.5		17.8±4.4				
	Progression rate(/years)	0.6±0.9		1.2±1.1		−0.03±1.2				
**Overlapping figure** ‡	Baseline (/40)	33.4±4.0		29.6±2.4		25.3±6.3		Non-converters>Memory-plus^b^		
	Progression rate(/years)	−0.2±1.0		0.7±0.9		−0.9±2.0				
**Backward digit-span** §	Baseline	4.4±0.8		3.0±0.9		4.0±0.7		Non-converters>Memory-only^a^		
	Progression rate(/years)	−0.1±0.3		0.1±0.4		−0.2±0.2				
**# of patients below −1 SD at baseline and at third year**	ADAS word recall †	9/26	3/26	3/7	1/7	3/6	3/6		NE	
	Overlapping figure‡	4/26	3/26	2/7	0/7	5/6	5/6		NE	
	Backward digit-span§	1/25	5/25	4/6	4/6	1/5	3/5		NE	

Analysis of variance with post-hoc Tukey’s test was used for group-wise comparisons of baseline scores and progression rates except for the UPDRS tremor/non-tremor scores. Two-way analysis of variance with post-hoc Tukey’s test was used for the UPDRS tremor/non-tremor scores. Data are given as the mean±SD except for the fields with asterisks. a and b indicate p<0.05 and p<0.01, respectively.

*Data are given as (the number of patients below −1 SD)/(the number of patients who underwent the test).

¶ The scores were calculated according to Lewis and colleagues. [5] Data were obtained from 21 non-converters, 6 memory-only converters and 5 memory-plus converters.

† The mean score for controls (n = 20, 65.5±4.8 years) is 21.3±3.5. [49].

‡ The mean score for controls (n = 24, 66.1±5.3 years) is 32.9±4.4. [32].

§ The mean score for controls (n = 20, 65.5±4.8 years) is 4.8±1.0. [49].

Abbreviations: UPDRS, Unified Parkinson's Disease Rating Scale; CDR, Clinical Dementia Rating; MMSE, Mini-Mental State Examination; ADAS, Alzheimer's Disease Assessment Scale; NE, not examined.

**Table 2:** Demographic and clinical profiles of patients with a Clinical Dementia Rating of 0.5 or more at baseline.

		Baseline memory-only (N = 8)		Baseline memory-plus (N = 6)		Differences between groups
**Age at baseline** (years)		69.0±6.6		66.2±5.5		
**Gender** (male/female)		6/2		6/0		
**Education** (years)		12.3±2.3		14.3±2.7		
**Test-retest interval** (days)		1115.3±107.1		1096.7±54.7		
**Disease duration at baseline** (years)		6.8±3.3		9.7±6.8		
**Age at onset** (years)		62.4±6.6		56.6±8.0		
**Levodopa equivalent dose at baseline** (mg/day)		453.6±163.1		658.6±337.9		
**UPDRS part III**	Baseline	27.1±5.4		23.8±6.6		
	Progression rate(/years)	−0.7±3.3		4.6±5.0		Baseline memory-plus>Baseline memory-only^a^
**UPDRS tremor** ¶	Baseline	0.7±0.5		0.4±0.6		
	Third year	0.3±0.2		0.4±0.7		Main effect of non-tremor score: Baseline memory-plus>Baseline memory-only^a^
**UPDRS non-tremor** ¶	Baseline	1.2±0.2		1.0±0.1		
	Third year	1.1±0.2		1.7±0.7		
**CDR sum of boxes**	Baseline	0.5		2.1±1.3		NE
	Third year	1.4±1.2		5.3±4.1		NE
**MMSE**	Baseline (/30)	27.0±3.0		27.0±2.2		
	Progression rate(/years)	−0.3±0.7		−1.1±2.7		
**ADAS word recall** †	Baseline (/30)	17.9±4.1		14.3±5.4		
	Progression rate(/years)	−0.1±1.4		−0.3±1.2		
**Overlapping figure** ‡	Baseline (/40)	29.6±4.1		29.4±6.2		
	Progression rate(/years)	0.1±1.1		−2.4±3.1		
**Backward digit-span** §	Baseline	3.6±1.0		3.8±0.5		NE
	Progression rate(/years)	−0.1±0.1		−0.3±0.3		NE
**# of patients below −1 SD at baseline and at third year**	ADAS word recall†	4/8	3/8	5/6	5/6	NE
	Overlapping figure‡	3/8	4/8	2/5	5/5	NE
	Backward digit-span§	3/7	4/7	1/4	2/4	NE

Two-sample *t*-tests were used for group-wise comparisons of baseline scores and progression rates except for the UPDRS tremor/non-tremor scores. A two-way analysis of variance was used for the UPDRS tremor/non-tremor scores. No group-wise comparisons were performed for the backward digit-span owing to the small number of subjects. Data are given as the mean±SD except for the fields with asterisks. a and b indicate p<0.05 and p<0.01, respectively.

*Data are given as (the number of patients below −1 SD)/(the number of patients who underwent the test).

¶ The scores were calculated according to Lewis and colleagues. [5] Data were obtained from 6 baseline memory-only and 6 baseline memory-plus patients.

† The mean score of controls (n = 20, 65.5±4.8 years) is 21.3±3.5. [49].

‡ The mean score of controls (n = 24, 66.1±5.3 years) is 32.9±4.4. [32].

§ The mean score of controls (n = 20, 65.5±4.8 years) is 4.8±1.0. [49]; a statistical comparison was not performed owing to an insufficient number of subjects.

Abbreviations: UPDRS, Unified Parkinson's Disease Rating Scale; CDR, Clinical Dementia Rating; MMSE, Mini-Mental State Examination; ADAS, Alzheimer's Disease Assessment Scale; NE, not examined.

### Statistical analyses

Group-wise comparisons of demographic data and baseline scores and progression rates of the cognitive and motor measures were analyzed using the statistical methods described in the captions of **Tables 1** **and** **2**. Two-way repeated-measures analysis of variance (ANOVA) with motor subtypes (the tremor and non-tremor scores of UPDRS) and time (baseline and third year) was performed to characterize the motor features of the groups. To enable comparisons with previous studies in which cognitive subtypes were determined by neuropsychological test scores, we investigated the number of patients whose scores were 1 SD or more below the mean of normative data for the ADAS-word recall, overlapping figure and backwards digit-span tests.

### 18F-fluorodeoxyglucose positron emission tomography

The mean interval between the clinical assessments and the positron emission tomography (PET) scan was 4.6 days. Each patient had fasted, and dopaminergic medication had been discontinued for at least 5 hours before the scan. Scanning was performed after an injection of 185–218 MBq 18F-fluorodeoxyglucose (FDG). After an FDG-uptake period of 1 hour, a 20-minute scan was acquired while the patient was at rest. Details of the scanning procedures have described elsewhere. [17], [32] Image pre-processing and statistical analysis were performed using SPM5 (http://www.fil.ion.ucl.ac.uk/spm/). All images were normalized onto the standard FDG template with nonlinear warping algorithms, reconstructed into 2 mm^3^ isotropic voxels and smoothed with 10 mm full width at half-maximum. Global normalisation was performed using proportional scaling, and threshold masking was set at 0.8. Cross-sectional comparisons between the patient groups and the controls were performed using *t*-test. Two-way repeated-measures ANOVA was used for cross-sectional and longitudinal comparisons of the patient groups. Age and sex were included as nuisance variables in all of the comparisons. The UPDRS part III score was included as a nuisance variable in the comparisons among the patient groups. The statistical threshold was set at an uncorrected p<0.001 at the voxel level and at 20 voxels at the cluster level.

## Results

### Comparison between the neuropsychology-based criteria and the Clinical Dementia Rating-based criteria

**Figure 1:**
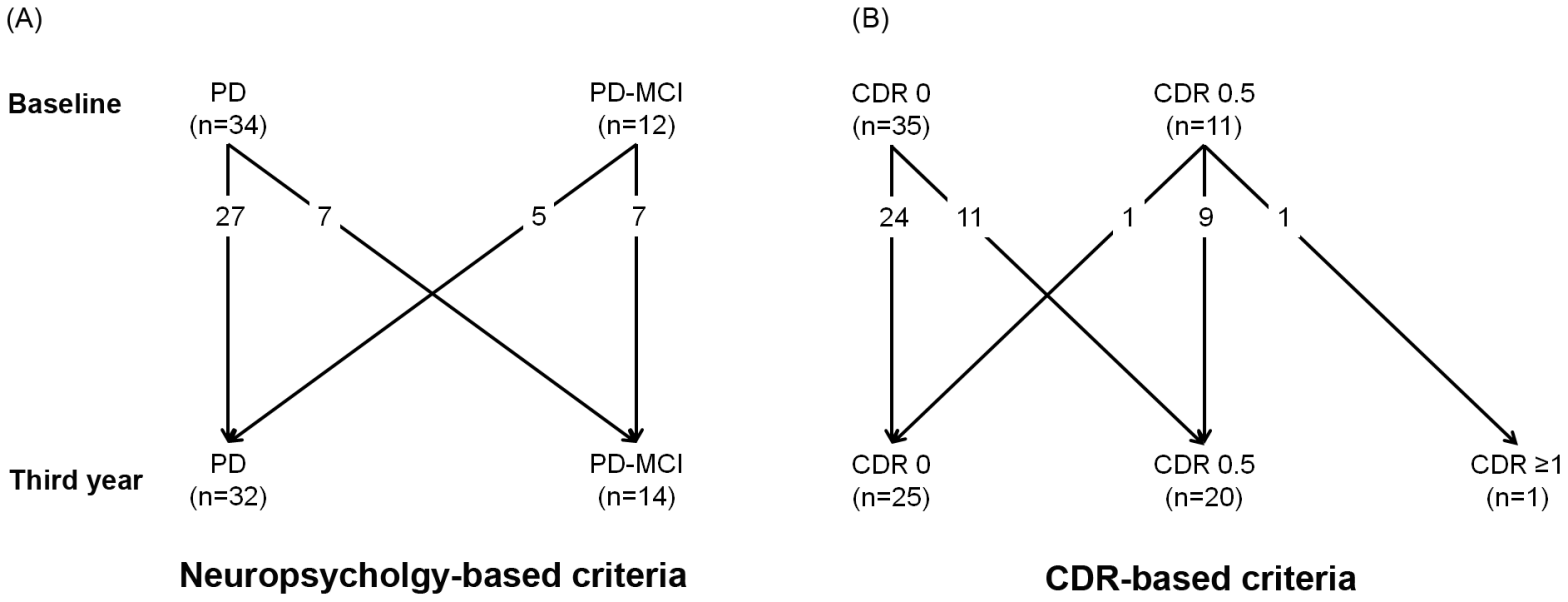
Diagrams of the 3-year cognitive changes observed in patients. In (A), the patients were classified as having Parkinson's disease without cognitive impairment (PD) or PD with mild cognitive impairment (PD-MCI) based on neuropsychological tests. (B) shows the results based on the Clinical Dementia Rating (CDR)-based patient classification.

The results are summarized in **Figure 1** **.** The neuropsychology-based classification according to the MDS PD-MCI criteria exhibited a spurious improvement over 3 years in 5 of the 12 patients who were classified to PD-MCI at baseline, whereas such an effect was observed only in 1 of the 11 patients who scored 0.5 on the baseline CDR (**Figure 1**). Based on these preliminary findings, we decided to employ the CDR-based cognitive criteria in the current study.

### Clinical profiles of the patient groups of baseline Clinical Dementia Rating 0

The results are summarized in **Table 1**. There were no significant differences among the non-converters, memory-only converters and memory-plus converters in sex, education, disease duration, levodopa equivalent dose or test-retest interval. The memory-plus converters had a significantly higher age of onset and a higher age at baseline than did the non-converters.

Baseline performance of the overlapping figure test was lower in the memory-plus converters than in the non-converters (F = 10.1, p<0.001). The baseline performance of the backwards digit-span was worse in the memory-only converters than it was in the non-converters (F = 7.1, p<0.01). No group differences were observed in baseline MMSE or baseline ADAS word recall. There were no significant differences in the progression rate on any of the cognitive tests.

No significant difference was observed in the baseline UPDRS part III among the three groups. The progression rate of the UPDRS part III was greater in the memory-plus converters than it was in the non-converters and the memory-only converters (F = 6.8, p<0.01). The UPDRS non-tremor score was higher in the memory-plus converters than it was in the other groups (F = 18.8, p<0.001), and no significant main effect of time or interaction between motor subtypes and times was observed.

### Clinical profiles of the patient groups of baseline Clinical Dementia Rating 0.5

The results are summarized in **Table 2**. There were no significant differences between the baseline memory-only and the baseline memory-plus patients in age at baseline, sex, education, age of onset, disease duration, levodopa equivalent dose or test-retest interval. No significant group differences were observed in the baseline scores or progression rates on any of the cognitive tests. No significant difference was observed in the baseline UPDRS part III score. The UPDRS part III progression rate was greater in the baseline memory-plus patients than it was in the baseline memory-only patients (t = −2.4, p<0.05). The UPDRS non-tremor score was higher in the baseline memory-plus patients than it was in the baseline memory-only patients (F = 8.0, p<0.001).

### Positron emission tomography: comparisons between patient groups and controls

**Figure 2:**
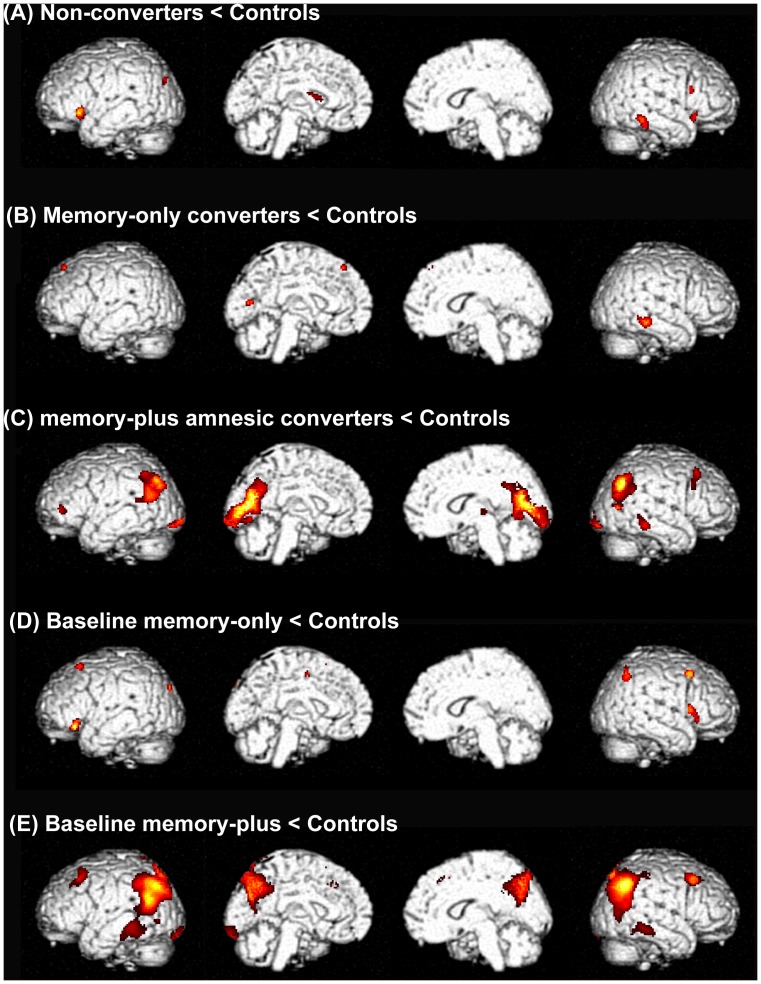
Areas of relative reduction in regional cerebral glucose metabolism in the patient groups compared with controls. Rendered images are shown in the order of the left lateral, left medial, right medial and right lateral.

Compared with the controls, the non-converters and memory-only converters exhibited patchy, discrete areas of hypometabolism in the frontal, temporal and occipital cortices at baseline (**Figures 2A and 2B**). The memory-plus converters showed extensive hypometabolic areas in the temporo-parietal and occipital cortices compared with the controls (**Figure 2C**).

The regional pattern of metabolic reduction relative to the controls was similar among the baseline memory-only patients, the non-converters and the memory-only converters (**Figure 2D**). The baseline memory-plus patients showed a similar but more extensive hypometabolism compared with the memory-plus converters, in whom the metabolic reduction relative to controls was greatest in the temporo-parietal and medial parietal cortices (**Figure 2E**).

### Positron emission tomography: comparisons among the patient groups of baseline Clinical Dementia Rating 0

**Figure 3:**
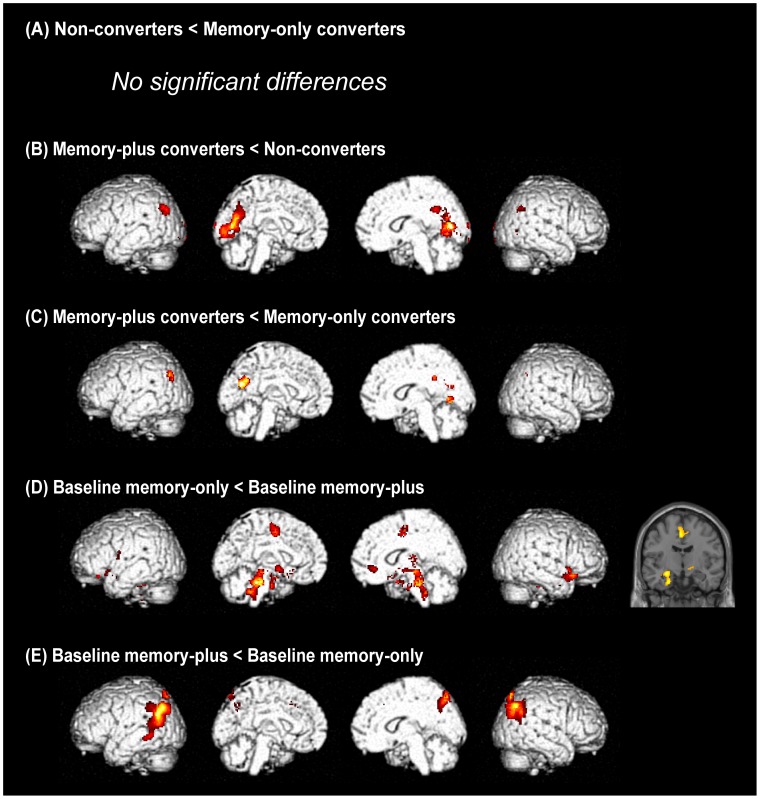
Group comparisons of regional cerebral glucose metabolism at baseline. (A) to (C) show the results of comparisons between patient groups with baseline Clinical Dementia Rating (CDR) 0, and (D) and (E) show the results of comparisons between groups with baseline CDR 0.5. Rendered images are shown in the order of the left lateral, left medial, right medial and right lateral. The left side of a coronal section corresponds to the left side of the brain.

At baseline, there was no significant difference in regional glucose metabolism between the non-converters and memory-only converters (**Figure 3A**). The memory-plus converters showed a stronger metabolic reduction in the parietal and occipital cortices compared with the non-converters and amnesic converters at baseline (**Figures 3B and 3C**).

**Figure 4:**
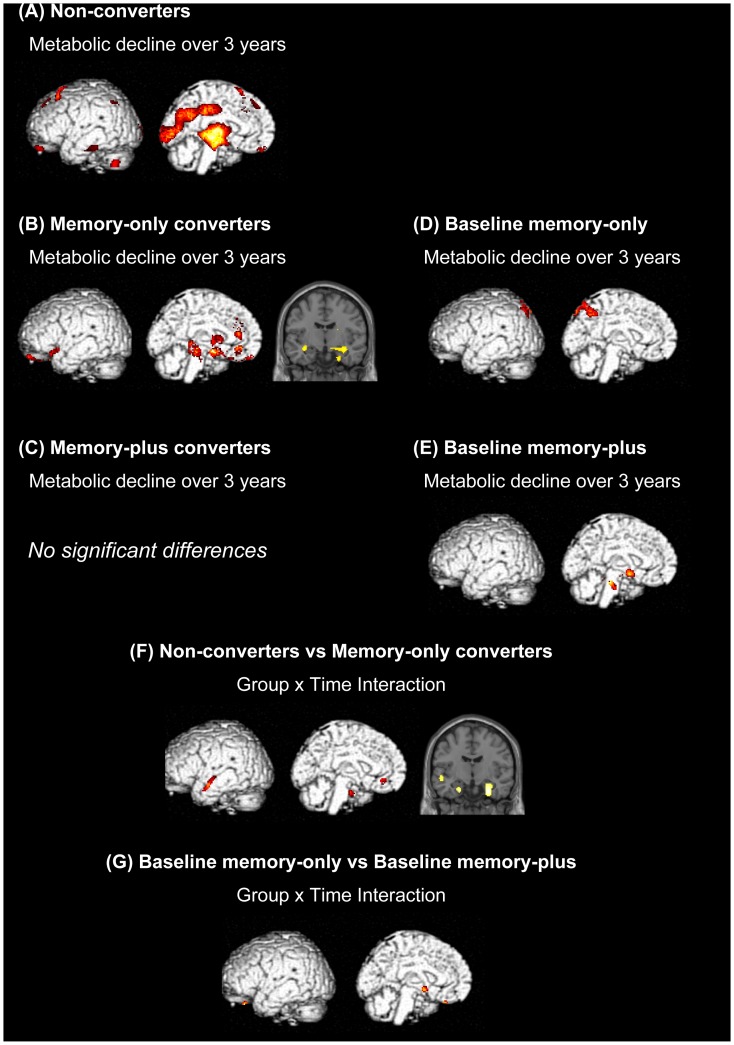
Longitudinal changes in regional cerebral glucose metabolism. (A) to (E) show 3-year metabolic declines in the individual patient groups. (F) and (G) show group x time interactions between the non-converters and the memory-only converters and between the baseline memory-only patients and the baseline memory-plus patients, respectively. Rendered images show the left hemisphere. The left sides of coronal sections correspond to the left side of the brain.

The non-converters showed a significant metabolic decline over 3 years in the frontal, temporal, medial parietal and occipital cortices and the thalamus (**Figure 4A**). In the memory-only converters, regional glucose metabolism was decreased in the anterior cingulate cortex, medial temporal lobe, caudate nucleus and midbrain over 3 years (**Figure 4B**). No significant longitudinal metabolic change was observed in the memory-plus converters (**Figure 4C**). An ANOVA interaction demonstrated that metabolic decline over 3 years in the medial temporal lobe was greater in the memory-only converters than it was in the non-converters (**Figure 4F**).

### Positron emission tomography: comparisons between the patient groups of baseline Clinical Dementia Rating 0.5

The baseline memory-only patients had lower baseline regional glucose metabolism in the medial temporal lobe, cingulate cortex and dorsal brainstem regions than did the baseline memory-plus patients, whereas the regional glucose metabolism in the temporo-parietal and medial parietal cortices was lower in the baseline memory-plus patients than it was in the baseline memory-only patients (**Figures 3D and 3E**).

Regional glucose metabolism was decreased over 3 years in the parietal cortex in the baseline memory-only patients, whereas a longitudinal metabolic decline was observed in discrete regions of the basal forebrain and the brainstem in the baseline memory-plus patients (**Figures 4D and 4E**). An ANOVA interaction revealed circumscribed ventral frontal and basal forebrain regions that showed a greater 3-year metabolic decline in the baseline memory-plus patients than in the baseline memory-only patients (**Figure 4G**).

## Discussion

### Early visuoperceptual impairment and posterior cortical hypometabolism may represent the clinical subtypes of rapidly progressive motor symptoms and severe cognitive impairment

The clinical entity of PD encompasses a wide variety of symptoms, including motor, sensory, cognitive and autonomic disturbances. Recent cluster-analysis studies have suggested that two major clinical subtypes can be extracted from the clinical diversity: one subtype is characterized by a young age of onset, slow disease progression, tremor-dominant motor features and preserved cognition, and the other is associated with an older age of onset, rapid disease progression, non-tremor-dominant motor features and cognitive impairment. [5], [6], [34] In parallel with these discoveries, there has been growing evidence of the neuropathological diversities underlying these clinical subtypes. Patients with a young age of onset, slow progression and tremor-dominant motor features are reported to have neuropathological features that conform to Braak's pathological staging scheme, in which Lewy-related pathology begins in the lower brainstem (stages 1–2); ascends to the midbrain (stage 3), thalamus and limbic structures (stages 4); and finally reaches the neocortex (stages 5–6). [35] By contrast, patients with an older age of onset, non-tremor-dominant motor features and/or dementia are associated with disproportionately severe neocortical Lewy-related pathology and concomitant Alzheimer's disease-related pathology. [14], [15].

In the current study, the memory-only converters showed a metabolic decline over 3 years in the anterior cingulate and medial temporal cortices (**Figure 4B**). The baseline memory-only patients, whose baseline cognitive status was similar to that of the memory-only converters at the third year, showed a metabolic decline in the parietal cortex (**Figure 4D**). Assuming that these patient groups represent a single cognitive subtype at different time points, these results suggest that neurodegeneration first affects the limbic structures and next encroaches on the posterior neocortex. This pattern of brain metabolic changes is largely consistent with Braak's scheme. [7] A longitudinal PET analysis of the non-converters demonstrated 3-year metabolic decline in the thalamus and occipital cortex (**Figure 4A**). A direct comparison between the non-converters and the memory-only converters revealed no significant group difference at baseline but greater metabolic decline over time in the memory-plus converters than in the non-converters (**Figures 3A** **and** **4F**). These two groups of patients may represent slightly different subpopulations of a clinico-pathological subtype that conforms to Braak's scheme.

The memory-plus converters exhibited extensive posterior cortical hypometabolism at baseline compared with the controls and the non-converters (**Figures 2C** **,** **3B and 3C**). Likewise, more extensive posterior cortical hypometabolism was observed in the baseline memory-plus patients compared with the baseline memory-only patients (**Figures 2E** **and** **3E**). These findings can be interpreted in two ways: the posterior neocortical hypometabolism found in these patients may represent pathological changes in Braak stages 5–6, or they may represent a pathological progression pattern that does not conform to Braak's scheme. [7] The latter was suggested by the following clinical and neuroimaging findings. First, the severity of motor symptoms at baseline was equivalent in the memory-plus converters, non-converters and memory-only converters, suggesting that the three groups had similar degrees of midbrain pathology. In other words, if the memory-plus converters represented a more advanced stage of the disease than did the other groups, they would not present with an equivalent severity of motor symptoms. Second, a comparison of metabolic patterns between the baseline memory-only and the baseline memory-plus patients showed a double dissociation in which posterior neocortical hypometabolism was more severe in the baseline memory-plus patients, whereas hypometabolism in the medial temporal lobe was more severe in the baseline memory-only patients (**Figures 3D and 3E**). These findings suggest that the brainstem and neocortex may be affected nearly simultaneously without marked limbic involvement in the memory-plus converters and the baseline memory-plus patients. A parallel finding was reported in a population-based cohort study in which incidental Lewy-related pathology was found in the brainstem and neocortex but not in the limbic structures (medial temporal and cingulate cortices) in 3% of cases. [36].

From the viewpoint of prediction and early intervention, it is critical to establish the cognitive and neuroimaging features that are associated with rapid symptomatic deterioration and the future development of dementia. [2] In the current study, the memory-plus converters exhibited clinical features that are consistent with those of the clinical subtype associated with the rapid progression of motor symptoms and/or dementia, including rapid declines in the CDR sum of boxes and the UPDRS part III scores, and non-tremor dominant motor features (**Table 1**). [4], [5], [6] They had impaired performance on the overlapping-figure test (**Table 1**) and posterior cortical hypometabolism at baseline (**Figures 2C** **,** **3B and 3C**), suggesting that early visuoperceptual impairment and posterior neocortical involvement may be risk factors for rapid symptomatic deterioration and the future development of dementia. The predictive value of visuoperceptual impairment for the future development of dementia in PD has been demonstrated in 3 of the 4 previous longitudinal neuropsychological studies with a follow-up of 2 years or more. [37], [38], [39], [40] Similarly, a recent study demonstrated that patients with non-amnestic multi-domain MCI that had visuoperceptual deficits were associated with bradykinesia and gait disturbance (non-tremor-dominant motor features), suggesting a link to the rapidly progressive, dementia-related clinico-pathological subtype. [41] Although there is no neuropathological evidence for the relationship between lesions in the particular cortical regions and rapid symptomatic progression and dementia in PD, a previous longitudinal FDG-PET study demonstrated that parieto-occipital hypometabolism preceded the development of dementia. [42].

### Memory impairment and its predictive value for future development of dementia in PD

Recent studies have demonstrated that memory impairment is the most common cognitive deficit in non-demented PD. [43], [44] In agreement with these findings, positive scores on the memory subdomain were the most commonly observed CDR findings and baseline impairment in the ADAS-word recall test was found in 45% of the patients in the current study (**Tables 1** **and** **2**). However, the results of the previous longitudinal neuropsychological studies were split regarding the predictive value of memory impairment for dementia in PD. [37], [38], [39], [40] One of the possible factors associated with this inconsistency is the variability of memory tests. The materials to be remembered (words, stories or figures) and the duration of retention (immediate or delayed) vary from test to test. Another possible factor which contribute to the low predictive value of memory impairment for dementia is the variability of the neural substrates of memory impairment in PD. Memory impairment in PD is associated with both dysexecutive retrieval deficits due to fronto-striatal dopaminergic insufficiency and mnemonic dysfunction due to hippocampal degeneration. [45] In the current study, baseline impairment on the backward digit-span observed in the memory-only converters suggests the possible contribution of executive/working memory deficits to memory complaints in PD (**Table 1**), whereas the relative medial temporal hypometabolism in the memory-only converters and the baseline memory-only patients suggested the role for hippocampal/medial temporal dysfunction (**Figures 3D** **,** **4B and 4F**). Furthermore, a third mechanism of memory impairment is indicated by the findings of the current study; the memory-plus converters and the baseline memory-plus patients did not show significant hypometabolism in the medial temporal lobe despite their obvious memory problems, but they instead showed temporo-parietal and medial parietal hypometabolism (**Figures 2C, 2E** **,** **3B, 3C and 3E**). The involvement of the parietal lobe in memory tasks has been documented in functional neuroimaging studies, but its functional role has been a matter of debate. [46].

### Limitations

There are a number of limitations in the current study. First, although we claim that the memory-plus converters represent the rapidly progressive clinical subtype, no significant metabolic changes over 3 years were observed in this patient group. The following reasons can be suggested for this negative finding: (1) the small sample size may have result in a low statistical power; and (2) diffuse metabolic decline across the entire cerebral cortex may have obscured by the proportional scaling in the PET analysis. Consistent with the latter, a supplementary PET analysis in which a cerebellar reference was used instead of the proportional scaling demonstrated a CMRglc reduction over 3 years in the preforntal cortex in the memory-plus converters (**Figure S1**).

Second, there were substantial inconsistencies between the CDR-based criteria and performance on the individual neuropsychological tests. Although patients with a CDR of 0 were defined as cognitively normal' according to our criteria, some were impaired in one or more neuropsychological tests. This inconsistency is most likely due to the insensitivity of the CDR to slight cognitive impairment, particularly in the executive and visuoperceptual domains. By contrast, neuropsychological tests failed to detect cognitive declines over time in the memory-only converters and memory-plus converters, despite the obvious cognitive deterioration documented by the CDR (**Table 1**). Measuring longitudinal cognitive changes using neuropsychological tests is contaminated by spurious improvement associated with practice effects. [20], [21], [22], [23] Although the neuropsychological tests were administered twice with a relatively long interval of 3 years in the current study, previous studies demonstrated that practice effects persist over 5 years and are strongest between the first and second administrations. [20], [47], [48] Furthermore, the impact of dopaminergic therapy on cognition and mood should be taken into account in PD patients. A formal definitions of clinically meaningful cognitive decline' in PD should be established in future studies. [29] In addition, the criteria for at-risk state for dementia or PD-MCI should be not only sensitive but also specific. Insensitive criteria would lead to the oversight of at-risk patients of dementia, whereas an overly sensitive and insufficiently specific ones would make every PD patient an at-risk one because almost every PD patient is impaired in some of highly-demanded cognitive tasks.

Third, we separately analyzed the patient groups with a baseline CDR of 0 and those with a CDR of 0.5 and integrated the results obtained from these separate analyses to discuss long-term (more than 3 years) cognitive changes. Our findings and discussion should be examined by studies with longer follow-up periods.

Finally, the reduction in glucose metabolism may reflect not only neurodegeneration itself but also the remote effects of lesions in distant neural structures. In addition, because FDG-PET is unable to differentiate between Alzheimer's disease-related and Lewy-related pathologies, further studies utilizing amyloid-PET and other neuroimaging techniques are necessary to examine these issues.

## Supporting Information

Figure S1 **The results of a cerebellar-referenced PET analysis for the patient groups with baseline CDR 0 (non-converters, memory-only converters and memory-plus converters).** A two-way repeated-measures ANOVA with variables of no interest of age, sex and UPDRS part III score was used. The statistical threshold was set at an uncorrected p<0.001 at the voxel level and at 20 voxels at the cluster level.
(TIF)

## References

[1] Kehagia AA, Barker RA, Robbins TW (2010) Neuropsychological and clinical heterogeneity of cognitive impairment and dementia in patients with Parkinson's disease. Lancet Neurol 9: 1200–1213. 20880750 10.1016/S1474-4422(10)70212-X

[2] Litvan I, Aarsland D, Adler CH, Goldman JG, Kulisevsky J, et al (2011) MDS Task Force on mild cognitive impairment in Parkinson's disease: critical review of PD-MCI. Mov Disord. 26: 1814–1824. 10.1002/mds.23823 PMC3181006 21661055

[3] Baba T, Kikuchi A, Hirayama K, Nishio Y, Hosokai Y, et al (2012) Severe olfactory dysfunction is a prodromal symptom of dementia associated with Parkinson's disease: a 3 year longitudinal study. Brain 135: 161–169. 22287381 10.1093/brain/awr321

[4] Burn DJ, Rowan EN, Allan LM, Molloy S, O'Brien JT, et al (2006) Motor subtype and cognitive decline in Parkinson's disease, Parkinson's disease with dementia, and dementia with Lewy bodies. J Neurol Neurosurg Psychiatry 77: 585–589. 16614017 10.1136/jnnp.2005.081711 PMC2117449

[5] Lewis SJ, Foltynie T, Blackwell AD, Robbins TW, Owen AM, et al (2005) Heterogeneity of Parkinson's disease in the early clinical stages using a data driven approach. J Neurol Neurosurg Psychiatry 76: 343–348. 15716523 10.1136/jnnp.2003.033530 PMC1739569

[6] van Rooden SM, Colas F, Martinez-Martin P, Visser M, Verbaan D, et al (2011) Clinical subtypes of Parkinson's disease. Mov Disord 26: 51–58. 21322019 10.1002/mds.23346

[7] Halliday GM, Holton JL, Revesz T, Dickson DW (2011) Neuropathology underlying clinical variability in patients with synucleinopathies. Acta Neuropathol 122: 187–204. 21720849 10.1007/s00401-011-0852-9

[8] Burton EJ, McKeith IG, Burn DJ, Williams ED, O'Brien JT (2004) Cerebral atrophy in Parkinson's disease with and without dementia: a comparison with Alzheimer's disease, dementia with Lewy bodies and controls. Brain 127: 791–800. 14749292 10.1093/brain/awh088

[9] Hosokai Y, Nishio Y, Hirayama K, Takeda A, Ishioka T, et al (2009) Distinct patterns of regional cerebral glucose metabolism in Parkinson's disease with and without mild cognitive impairment. Mov Disord 24: 854–862. 19199357 10.1002/mds.22444

[10] Huang C, Mattis P, Tang C, Perrine K, Carbon M, et al (2007) Metabolic brain networks associated with cognitive function in Parkinson's disease. Neuroimage 34: 714–723. 17113310 10.1016/j.neuroimage.2006.09.003 PMC4456012

[11] Nagano-Saito A, Washimi Y, Arahata Y, Kachi T, Lerch JP, et al (2005) Cerebral atrophy and its relation to cognitive impairment in Parkinson disease. Neurology 64: 224–229. 15668417 10.1212/01.WNL.0000149510.41793.50

[12] Summerfield C, Junque C, Tolosa E, Salgado-Pineda P, Gomez-Anson B, et al (2005) Structural brain changes in Parkinson disease with dementia: a voxel-based morphometry study. Arch Neurol 62: 281–285. 15710857 10.1001/archneur.62.2.281

[13] Aarsland D, Perry R, Brown A, Larsen JP, Ballard C (2005) Neuropathology of dementia in Parkinson's disease: a prospective, community-based study. Ann Neurol 58: 773–776. 16240351 10.1002/ana.20635

[14] Compta Y, Parkkinen L, O'Sullivan SS, Vandrovcova J, Holton JL, et al (2011) Lewy- and Alzheimer-type pathologies in Parkinson's disease dementia: which is more important? Brain 134: 1493–1505. 21596773 10.1093/brain/awr031 PMC4194668

[15] Selikhova M, Williams DR, Kempster PA, Holton JL, Revesz T, et al (2009) A clinico-pathological study of subtypes in Parkinson's disease. Brain 132: 2947–2957. 19759203 10.1093/brain/awp234

[16] Bohnen NI, Muller ML (2013) In vivo neurochemical imaging of olfactory dysfunction in Parkinson's disease. J Neural Transm 120: 571–576. 23263541 10.1007/s00702-012-0956-y PMC3612386

[17] Nishio Y, Hirayama K, Takeda A, Hosokai Y, Ishioka T, et al (2010) Corticolimbic gray matter loss in Parkinson's disease without dementia. Eur J Neurol 17: 1090–1097. 20298422 10.1111/j.1468-1331.2010.02980.x

[18] Amrerical Psychiatric Association. (1987) Diagnostic and Statistical Manual of Mental Disorders. 3rd, revised.

[19] Morris JC (1997) Clinical dementia rating: a reliable and valid diagnostic and staging measure for dementia of the Alzheimer type. Int Psychogeriatr 9 Suppl 1 173–176 discussion 177–178. 9447441 10.1017/s1041610297004870

[20] Abner EL, Dennis BC, Mathews MJ, Mendiondo MS, Caban-Holt A, et al (2012) Practice effects in a longitudinal, multi-center Alzheimer's disease prevention clinical trial. Trials 13: 217. 23171483 10.1186/1745-6215-13-217 PMC3543284

[21] Duff K, Beglinger LJ, Schultz SK, Moser DJ, McCaffrey RJ, et al (2007) Practice effects in the prediction of long-term cognitive outcome in three patient samples: a novel prognostic index. Arch Clin Neuropsychol 22: 15–24. 17142007 10.1016/j.acn.2006.08.013 PMC1847360

[22] Machulda MM, Pankratz VS, Christianson TJ, Ivnik RJ, Mielke MM, et al (2013) Practice Effects and Longitudinal Cognitive Change in Normal Aging vs. Incident Mild Cognitive Impairment and Dementia in The Mayo Clinic Study of Aging. Clin Neuropsychol 27: 1247–1264. 24041121 10.1080/13854046.2013.836567 PMC3869900

[23] Mathews M, Abner E, Caban-Holt A, Kryscio R, Schmitt F (2013) CERAD practice effects and attrition bias in a dementia prevention trial. Int Psychogeriatr 25: 1115–1123. 23570673 10.1017/S1041610213000367 PMC4012228

[24] Mathews M, Abner E, Kryscio R, Jicha G, Cooper G, et al. (2014) Diagnostic accuracy and practice effects in the National Alzheimer's Coordinating Center Uniform Data Set neuropsychological battery. Alzheimers Dement. doi:10.1016/j.jalz.2013.11.007. 10.1016/j.jalz.2013.11.007 PMC4169759 24656850

[25] Doody RS, Ferris SH, Salloway S, Sun Y, Goldman R, et al (2009) Donepezil treatment of patients with MCI: a 48-week randomized, placebo-controlled trial. Neurology 72: 1555–1561. 19176895 10.1212/01.wnl.0000344650.95823.03

[26] Dubois B, Tolosa E, Katzenschlager R, Emre M, Lees AJ, et al (2012) Donepezil in Parkinson's disease dementia: a randomized, double-blind efficacy and safety study. Mov Disord 27: 1230–1238. 22915447 10.1002/mds.25098

[27] Mori E, Ikeda M, Kosaka K (2012) Donepezil for dementia with Lewy bodies: a randomized, placebo-controlled trial. Ann Neurol 72: 41–52. 22829268 10.1002/ana.23557 PMC3504981

[28] Rogers SL (1998) Perspectives in the management of Alzheimer's disease: clinical profile of donepezil. Dement Geriatr Cogn Disord 9 Suppl 3 29–42. 10.1159/000051201 9853200

[29] Litvan I, Goldman JG, Troster AI, Schmand BA, Weintraub D, et al (2012) Diagnostic criteria for mild cognitive impairment in Parkinson's disease: Movement Disorder Society Task Force guidelines. Mov Disord 27: 349–356. 22275317 10.1002/mds.24893 PMC3641655

[30] Folstein MF, Robins LN, Helzer JE (1983) The Mini-Mental State Examination. Arch Gen Psychiatry 40: 812. 6860082 10.1001/archpsyc.1983.01790060110016

[31] Mohs RC, Rosen WG, Davis KL (1983) The Alzheimer's disease assessment scale: an instrument for assessing treatment efficacy. Psychopharmacol Bull 19: 448–450. 6635122

[32] Ishioka T, Hirayama K, Hosokai Y, Takeda A, Suzuki K, et al (2011) Illusory misidentifications and cortical hypometabolism in Parkinson's disease. Mov Disord 26: 837–843. 21370270 10.1002/mds.23576

[33] Schrag A, Dodel R, Spottke A, Bornschein B, Siebert U, et al (2007) Rate of clinical progression in Parkinson's disease. A prospective study. Mov Disord 22: 938–945. 17415791 10.1002/mds.21429

[34] Reijnders JS, Ehrt U, Lousberg R, Aarsland D, Leentjens AF (2009) The association between motor subtypes and psychopathology in Parkinson's disease. Parkinsonism Relat Disord 15: 379–382. 18977165 10.1016/j.parkreldis.2008.09.003

[35] Braak H, Del Tredici K, Rub U, de Vos RA, Jansen Steur EN, et al (2003) Staging of brain pathology related to sporadic Parkinson's disease. Neurobiol Aging 24: 197–211. 12498954 10.1016/s0197-4580(02)00065-9

[36] Zaccai J, Brayne C, McKeith I, Matthews F, Ince PG (2008) Patterns and stages of alpha-synucleinopathy: Relevance in a population-based cohort. Neurology 70: 1042–1048. 18362284 10.1212/01.wnl.0000306697.48738.b6

[37] Janvin CC, Larsen JP, Aarsland D, Hugdahl K (2006) Subtypes of mild cognitive impairment in Parkinson's disease: progression to dementia. Mov Disord 21: 1343–1349. 16721732 10.1002/mds.20974

[38] Levy G, Jacobs DM, Tang MX, Cote LJ, Louis ED, et al (2002) Memory and executive function impairment predict dementia in Parkinson's disease. Mov Disord 17: 1221–1226. 12465060 10.1002/mds.10280

[39] Mahieux F, Fenelon G, Flahault A, Manifacier MJ, Michelet D, et al (1998) Neuropsychological prediction of dementia in Parkinson's disease. J Neurol Neurosurg Psychiatry 64: 178–183. 9489527 10.1136/jnnp.64.2.178 PMC2169963

[40] Williams-Gray CH, Foltynie T, Brayne CE, Robbins TW, Barker RA (2007) Evolution of cognitive dysfunction in an incident Parkinson's disease cohort. Brain 130: 1787–1798. 17535834 10.1093/brain/awm111

[41] Goldman JG, Weis H, Stebbins G, Bernard B, Goetz CG (2012) Clinical differences among mild cognitive impairment subtypes in Parkinson's disease. Mov Disord 27: 1129–1136. 22778009 10.1002/mds.25062 PMC3412930

[42] Bohnen NI, Koeppe RA, Minoshima S, Giordani B, Albin RL, et al (2011) Cerebral glucose metabolic features of Parkinson disease and incident dementia: longitudinal study. J Nucl Med 52: 848–855. 21571793 10.2967/jnumed.111.089946 PMC7486964

[43] Aarsland D, Bronnick K, Williams-Gray C, Weintraub D, Marder K, et al (2010) Mild cognitive impairment in Parkinson disease: a multicenter pooled analysis. Neurology 75: 1062–1069. 20855849 10.1212/WNL.0b013e3181f39d0e PMC2942065

[44] Hanna-Pladdy B, Jones K, Cabanban R, Pahwa R, Lyons KE (2013) Predictors of mild cognitive impairment in early-stage Parkinson's disease. Dement Geriatr Cogn Dis Extra 3: 168–178. 23741229 10.1159/000351421 PMC3670639

[45] Aarsland D, Bronnick K, Fladby T (2011) Mild cognitive impairment in Parkinson's disease. Curr Neurol Neurosci Rep 11: 371–378. 21487730 10.1007/s11910-011-0203-1

[46] Wagner AD, Shannon BJ, Kahn I, Buckner RL (2005) Parietal lobe contributions to episodic memory retrieval. Trends Cogn Sci 9: 445–453. 16054861 10.1016/j.tics.2005.07.001

[47] Rabbitt P, Lunn M, Ibrahim S, McInnes L (2009) Further analyses of the effects of practice, dropout, sex, socio-economic advantage, and recruitment cohort differences during the University of Manchester longitudinal study of cognitive change in old age. Q J Exp Psychol (Hove) 62: 1859–1872. 19214831 10.1080/17470210802633461

[48] Rabbitt P, Lunn M, Wong D, Cobain M (2008) Age and ability affect practice gains in longitudinal studies of cognitive change. J Gerontol B Psychol Sci Soc Sci 63: P235–P240. 18689765 10.1093/geronb/63.4.p235

[49] Abe N, Fujii T, Hirayama K, Takeda A, Hosokai Y, et al (2009) Do parkinsonian patients have trouble telling lies? The neurobiological basis of deceptive behaviour. Brain 132: 1386–1395. 19339257 10.1093/brain/awp052 PMC2677797

