# Supplementary figures and images for: Neural Substrates of Cognitive Subtypes in Parkinson's Disease: A 3-Year Longitudinal Study
Source: PLoS One. 2014 Oct 20;9(10):e110547. doi: 10.1371/journal.pone.0110547 (PMC4203806; doi:10.1371/journal.pone.0110547)

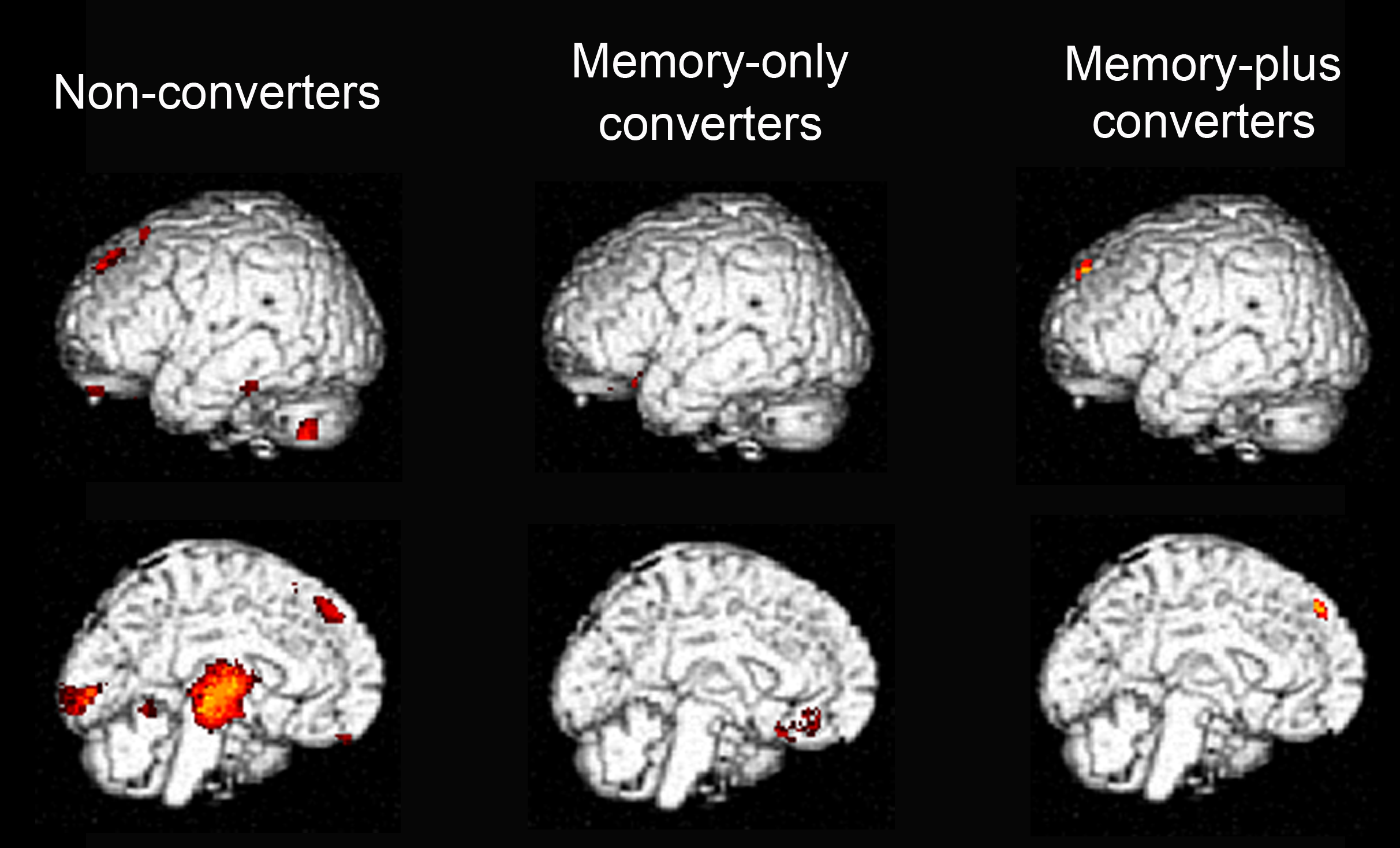

Supplement: Figure S1 — The results of a cerebellar-referenced PET analysis for the patient groups with baseline CDR 0 (non-converters, memory-only converters and memory-plus converters). A two-way repeated-measures ANOVA with variables of no interest of age, sex and UPDRS part III score was used. The statistical threshold was set at an uncorrected p<0.001 at the voxel level and at 20 voxels at the cluster level. (TIF) [file pone.0110547.s001.tif]
